# A multi-view fusion lightweight network for CRSwNPs prediction on CT images

**DOI:** 10.1186/s12880-024-01296-3

**Published:** 2024-05-16

**Authors:** Jisheng Zou, Yi Lyu, Yu Lin, Yaowen Chen, Shixin Lai, Siqi Wang, Xuan Zhang, Xiaolei Zhang, Renhua Wu, Weipiao Kang

**Affiliations:** 1 College of Engineering, Shantou University, Shantou, 515063 China; 2 Department of Otolaryngology, the Second Affiliated Hospital of Shantou University Medical College, Shantou, 515041 China; 3 Department of Radiology, the Second Affiliated Hospital of Shantou University Medical College, Shantou, 515041 China

**Keywords:** CT, Deep learning, Intrinsic type, Chronic sinusitis

## Abstract

Accurate preoperative differentiation of the chronic rhinosinusitis (CRS) endotype between eosinophilic CRS (eCRS) and non-eosinophilic CRS (non-eCRS) is an important topic in predicting postoperative outcomes and administering personalized treatment. To this end, we have constructed a sinus CT dataset, which comprises CT scan data and pathological biopsy results from 192 patients of chronic rhinosinusitis with nasal polyps (CRSwNP), treated at the Second Affiliated Hospital of Shantou University Medical College between 2020 and 2022. To differentiate CRSwNP endotype on preoperative CT and improve efficiency at the same time, we developed a multi-view fusion model that contains a mini-architecture with each network of 10 layers by modifying the deep residual neural network. The proposed model is trained on a training set and evaluated on a test set. The multi-view deep learning fusion model achieved the area under the receiver-operating characteristics curve (AUC) of 0.991, accuracy of 0.965 and F1-Score of 0.970 in test set. We compared the performance of the mini-architecture with other lightweight networks on the same Sinus CT dataset. The experimental results demonstrate that the developed ResMini architecture contribute to competitive CRSwNP endotype identification modeling in terms of accuracy and parameter number.

## Introduction

Chronic rhinosinusitis (CRS) is a prevalent chronic inflammatory condition of the upper respiratory tract that impacts individuals across all age demographics. The estimated prevalence rates of the condition are reported to be 12.3% in the United States, 10.9% in Europe, and 13% in China [1]. In clinical medicine, based on its association with nasal polyps CRS is classified into two categories: chronic rhinosinusitis without nasal polyps (CRSsNP) and chronic rhinosinusitis with nasal polyps (CRSwNP). However, this clinical categorization is no longer sufficient to address the growing complexity of clinical requirements. Particularly, the proposal of the “endotype” concept has emerged in light of the disclosure of the physiopathological mechanisms underlying CRS. This concept pertains to a subtype of disease that is characterized by functional and pathology attributes, which are determined by cellular, molecular, and immunological systems [2]. At present, there is a lack of standardized classification system for CRS endotype. Both domestic and international clinical guidelines commonly categorize it into two endotypes: eosinophilic CRS (eCRS) and non-eosinophilic CRS (non-eCRS). This classification is based on the predominant inflammatory cell types observed in histopathological analysis [3]. In the field of clinical practice, it is observed that various endotypes exhibit distinct diagnostic and treatment approaches [4]. Functional endoscopic sinus surgery (FESS), which prioritizes the preservation of mucosal tissue, is considered more appropriate for cases with non-eCRS. However, for eCRS cases characterized by a significant inflammatory burden, achieving favorable outcomes with FESS poses challenges [5]. It highlights the need of accurately diagnosing the endotype of CRS to facilitate the development of personalized and targeted treatment approaches.

**Fig. 1:**
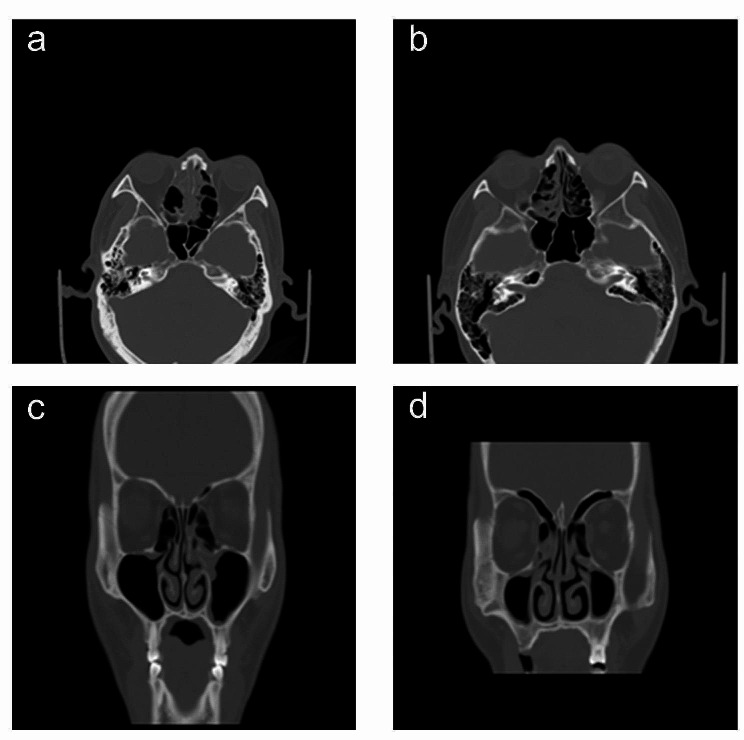
Computed tomography sections in axial (**a** and **b**) and coronal (**b** and **d**) planes of eCRS (**a** and **c**) and non-eCRS (**b** and **d**). eCRS exhibits predominant involvement in bilateral anterior and posterior ethmoid sinuses, whereas non-eCRS predominantly affects the anterior ethmoid sinuses

Currently, the conventional practice for detecting endotype primarily relies on pathologic biopsy, which is commonly regarded as the benchmark method. However, this approach is associated with certain drawbacks: (1) The procedure is considered invasive in nature. (2) Its capability is limited to acquiring information solely pertaining to inflammation in localized tissues, thereby resulting in a certain degree of misdiagnosis. (3) The procedure is exclusively applicable to patients’ undergoing surgery and can only be performed post-surgery, rendering it incapable of determining inflammation and ascertaining the endotype during the surgical and perioperative phases. Hence, the primary concern in the clinical domain of CRS precision diagnosis and treatment revolves around the accurate identification of the endotype of CRS prior to surgical intervention. The utilization of sinus computed tomography (CT) images is essential in objectively evaluating the severity of CRS patients, monitoring the effectiveness of treatment, and developing personalized and accurate surgical approaches prior to surgery. In practice, sinus CT is mainly used to evaluate eCRS and non-eCRS through LM score and GOSS score, where olfactory clefts (OC), posterior ethmoid (PE), ethmoid sinus/maxillary sinus (E/M) and ethmoid osteitis index are commonly used indicators, which are all obtained from CT images of the sinuses through manual interpretation. For patients with eCRS, CT findings predominantly indicate bilateral involvement of the anterior and posterior sigmoid sinuses, while non-eCRS typically exhibits predominant involvement of the anterior sigmoid sinus (Fig. 1). The evaluation systems most frequently utilized in sinus CT image analysis are the Lund-Mackay (LM) scoring system and the global osteitis scoring scale (GOSS) osteitis scoring system. Several researchers have employed the LM scoring system to forecast the inherent nature of CRS. Their findings indicate that the LM scoring system scores of patients with eCRS were notably higher compared to those patients with non-eCRS. Furthermore, the combination of an OC score greater than 1 and a PE score greater than 1 demonstrated the highest level of accuracy in predicting eCRS [6, 7]. Meng et al. employed the ratio of ethmoidal/maxillary sinus scores (E/M) ratio from the LM scoring system to assess the variability between eCRS and non-eCRS. The findings indicated that a score ratio of 2.0 and particularly 2.59 gave a high level of predicted accuracy [8]. The utilization of the GOSS osteitis scoring system was employed to predict the endotype of CRS. The findings of this study indicated that the ethmoidal osteitis score could serve as a reliable assessment metric for distinguishing between eCRS and non-eCRS. Specifically, when the score surpasses 4.5, eCRS can be preliminarily diagnosed through clinical means [9]. Furthermore, Zhu et al. have recently employed sinus CT imaging radiomics as a means to forecast eCRSwNP, achieving a commendable accuracy rate of 77.6% [10]. Nevertheless, there exist several limitations in the aforementioned evaluation techniques: (1) The LM scoring system, the GOSS osteitis scoring system, and the sinus CT imaging histology all involve manual customization for the extraction of image features. However, this manual approach introduces subjective factors that hinder the development of accurate prediction models. As a result, these models have remained in shallow feature learning research stage of the traditional machine learning, leading to suboptimal overall prediction performance. (2) The prediction and assessment methods discussed in this study lack prospective external data validation, which poses challenges in determining their clinical application value and hampers their widespread adoption in clinical settings. Additionally, the level of data refinement in these methods is relatively low, as they primarily rely on visual and quantitative analysis at a macroscopic level. Consequently, the identification of numerous potential microscopic image features remains challenging. Hence, the task of overcoming the limitations of superficial macroscopic observations and achieving intelligent and accurate prediction of the endotype of CRS has emerged as a fundamental scientific challenge that requires resolution in clinical settings.

Sinus CT images have a multitude of intricate microscopic features that are high-dimensional in nature. These features have the capacity to unveil underlying pathophysiological information embedded within the images. The robust feature extraction and screening capabilities of deep learning technology enable it to automatically capture and merge important picture features within complicated images that possess microscopic high-throughput and multi-channel data. Wu et al. retrospectively collected chest CT images from 495 patients in three hospitals in China. Based on deep learning methods, they proposed a multi-view image pneumonia diagnosis model using chest CT images [11]. However, there are limited research findings on the prediction of CRS intrinsic types based on nasal sinus CT images using deep learning techniques. Hua et al. employed U-net and other neural networks to construct a predictive model for CRS intrinsic types based on nasal sinus CT images [12]. Although achieving favorable accuracies 76.2% and 85.3% in predicting both image intrinsic type labels and patient intrinsic type labels, their study was constrained by the depth of the U-net neural network and the width of single-channel data, relying solely on axial plane nasal sinus CT images. Such limitations may have resulted in lower accuracy in image annotation and lacked validation with prospective data, thus hindering clinical applicability. Du et al. employed ResNet-18 to distinguish and predict the intrinsic types of CRSwNP, achieving a high AUC value on the test set [13]. However, their study did not fully utilize the information from different sections of CT scans from the same patient, potentially leading to information wastage. The backbone networks for classification, like ResNet [14], demonstrate exceptional performance across diverse huge datasets. However, they encounter drawbacks in real medical translational applications due to an excessive number of parameters and duplicated network structure. In response to the aforementioned scenario, we have made enhancements to the ResNet feature extraction module. In practice, we introduced a novel network architecture called ResNet mini architecture, which was employed for the purpose of extracting features from three-orientation CT images (axial, coronal, and sagittal). These features are utilized in the diagnosis of eosinophilic and non-eosinophilic CRSwNP.

## Materials and methods

### Datasets

This study received approval from the Institutional Review Board of the Second Affiliated Hospital of Shantou University Medical College. The members of the institutional review board granted an exemption for obtaining written consent for the right-to-know and provided approval for the retrospective study.

To establish a high-quality biospecimen bank of CRSwNP, we recruited patients with CRSwNP from the Second Affiliated Hospital of Shantou University Medical College. The inclusion criteria [15] were as follows: meeting the diagnostic criteria for CRSwNP as defined by previous research, no history of previous nasal surgery, normal liver and renal function, and no use of oral glucocorticoid medication or macrolide antimicrobials within 1 month prior to treatment. The exclusion criteria were as follows: being under 18 years old, having fungal sinusitis, posterior nostril polyps, nasal and sinus tumors, primary ciliary dyskinesia, cystic fibrosis, no sinus CT scan, and no previous sinus surgical treatment.

A total of 192 patients diagnosed with CRSwNP were included in this retrospective study. We collected sinus CT images from these patients, comprising three views: axial, coronal, and sagittal. In total, there were 21,108 images obtained, which were further categorized into two groups: eCRS (72 patients, 9,062 images) and non-eCRS (120 patients, 13,203 images). The image labels were assigned based on postoperative pathological endotypes. The dataset was partitioned into training and testing sets with an 4:1 ratio. Specifically, the training set encompassed 5628 data groups, which amounted to 16,884 CT images, while the testing set comprised 1408 data groups, totaling 4224 CT images. Each data group encapsulated three images: axial, coronal, and sagittal planes. Each image in the dataset possesses a certain resolution of 224 × 224. Furthermore, all images inside the dataset are represented in grayscale, with pixel intensities ranging from 0 to 255. Ultimately, the dataset is employed for the purposes of training and testing deep learning networks.

### Network architecture

#### Multi-view fusion deep learning architecture

In this study, we selected three-view pictures of sinus CT scans as the input for our model. The research conducted on the identification of lung nodules from chest CT images has demonstrated that deep learning models with a multi-view fusion technique exhibit superior performance compared to single-channel models [16]. Compared with the single channel model, the multi-view model is superior to the single channel model from the information point of view, because it uses more image information. The advantage of multi-view fusion model is to make full use of the information from different perspectives of CT scan and reduce the waste of information. Motivated by this, we employed CT images of the sinus region in axial, coronal, and sagittal planes as inputs to a deep neural network. This approach aims to enhance the diagnostic capabilities for identifying the endotype of CRSwNP by using information from many channels in a more complete and precise manner.

**Fig. 2:**
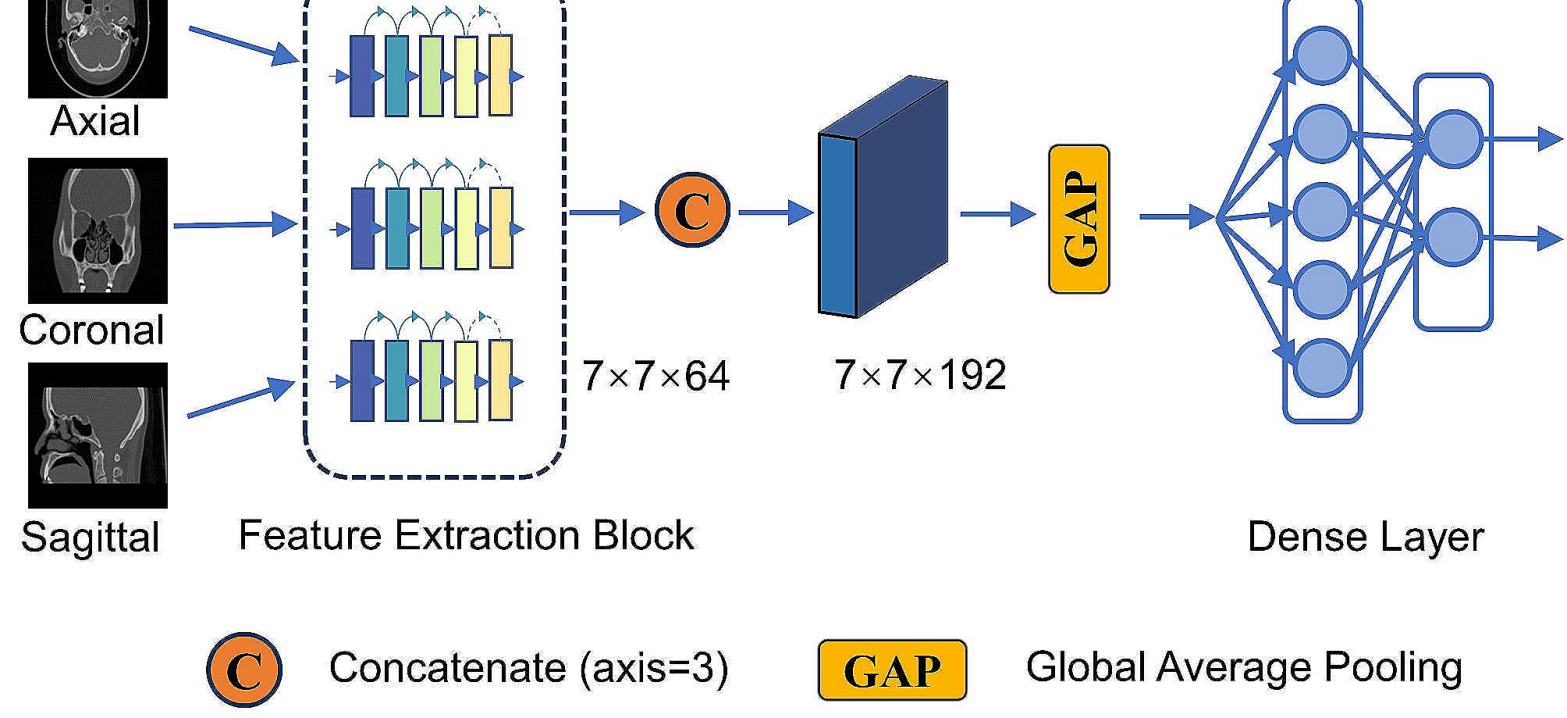
The main framework of multi-view deep learning fusion model. Three images in the axial, coronal, and sagittal planes are utilized as input. Subsequently, the output probability is mapped by a Dense layer after undergoing feature extraction and feature fusion operations

To determine the endotype of CRSwNP, a multi-view fusion network was devised utilizing a convolutional neural network, as depicted in Fig. 2. The network is comprised of four components, namely the input layer, feature extraction layer, feature fusion layer, and classification output layer. The set of axial, coronal, and sagittal CT images were partitioned into three channels for input into the network. The feature extraction component of the network was developed by adapting the ResNet-18 architecture, a residual network that incorporates a residual module. This module offers improved computational efficiency and parameter reduction without compromising the network’s accuracy. The three branch networks generate their respective feature maps, which are combined using the concatenate function and fed into the global average pooling layer to concentrate the features. These features are then passed into the fully connected layer, which ultimately produces the probability values for the different categories of CRSwNP endotype.

#### ResMini block

**Fig. 3:**
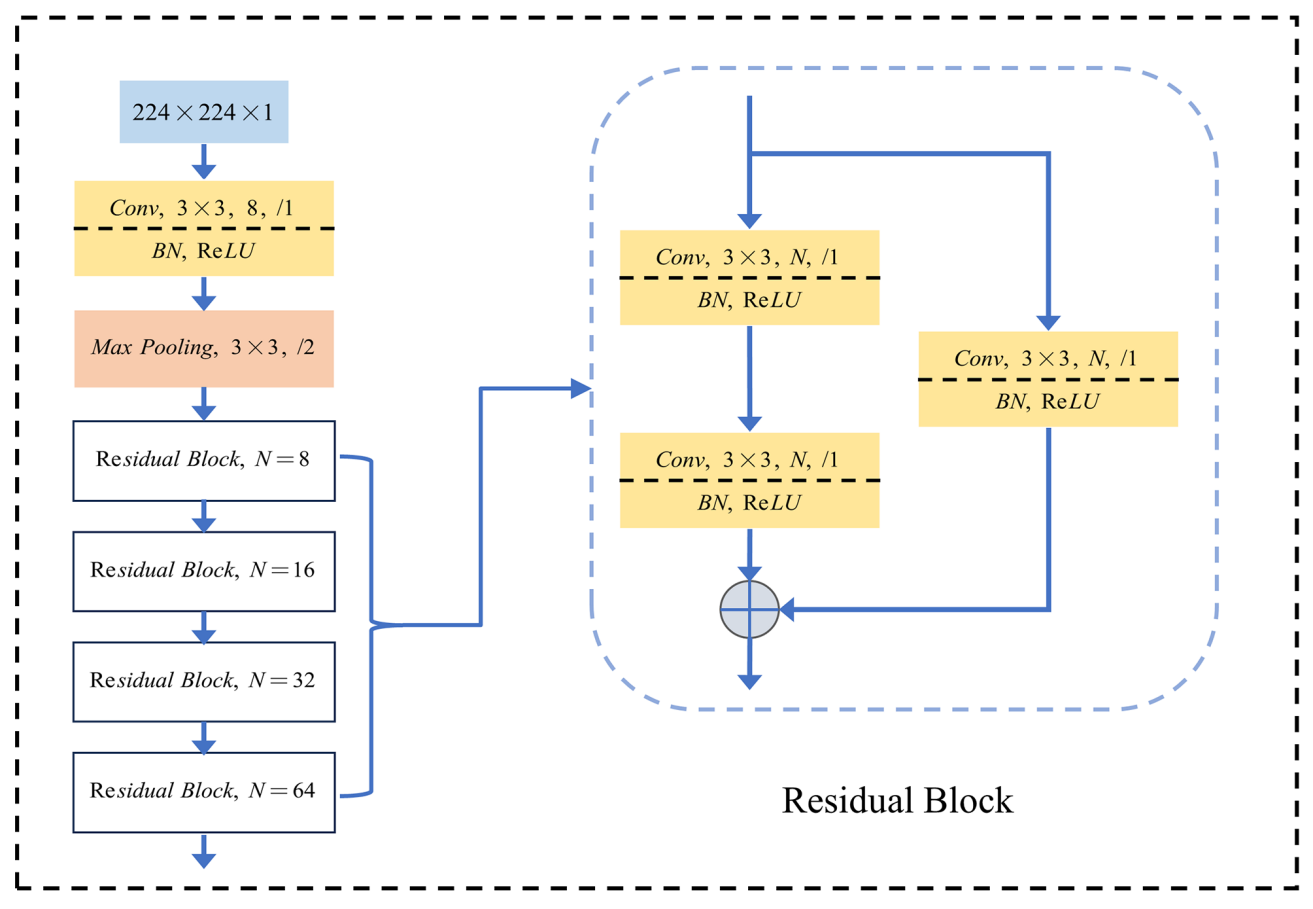
ResMini architecture

ResNet has demonstrated exceptional performance as a backbone network in image classification tasks, particularly on extensive datasets like ImageNet [17]. However, in specific contexts where efficiency is crucial for medical image classification tasks, such as disease screening or resource-constrained scenarios like embedded devices, ResNet may exhibit redundancy in terms of model size, with parameters exceeding the magnitude of ten million. In this article, we develop a modified ResNet architecture, in which the feature extraction module ResMini [18] is also referred to as the Residual Block, as depicted in Fig. 2. The ResMini architecture is specifically designed to have a depth of 10 layers, in contrast to ResNet-18 which has 18 layers. This deliberate design choice results in a significant reduction in network parameters while simultaneously enhancing overall performance. The left side of Fig. 3 illustrates the architectural composition of ResMini. Initially, the input undergoes processing through a CBA module, consisting of Convolution2D, Batch normalization, and Rectified Linear Unit (ReLU) activation function [19, 20]. Subsequently, the output is directed to a Max Pooling layer, immediately followed by the integration of four residual structures. The outputs of the residual modules from each branch are concatenated and subsequently fed into an Average Pooling layer and a Dense layer. Finally, the resulting output is sent via a Softmax function. The quantity of filters within the convolutional layers of the residual architecture increases twofold with the progression of the network’s depth.

In the CBA module, the kernel size of the convolutional layer is determined to be 3 × 3, with a stride of 1 and padding of 3. Additionally, the Max Pooling layer is configured with a Kernel size of 3 × 3, a stride of 2, and padding of 1. Following the convolutional layer, the utilization of Batch Normalization is employed to enhance the rate of convergence of the network, while the activation function of choice is Rectified Linear Unit (ReLU). The activation function employed in this context is ReLU.

The residual structure is depicted on the right side of Fig. 3. It consists of two branches: one branch comprises two CBA modules, while the other branch directly passes through a single CBA module. Ultimately, these two branches converge and produce the final output.

### Image processing and parameter setting

To enhance the model’s generalization capability, we conducted data augmentation such as panning, flipping, rotating, scaling, and cropping, as well as feature normalization on the dataset. Ultimately, the deep learning network was trained using the designated training set, and subsequently the network’s performance was assessed using the designated test set. The network model’s parameters update during the training phase through Adam optimizer [21]. The Cross Entropy Loss [22] was employed as the loss function. The learning rate was established at 1 × 10^− 5^, and a batch size of 20 was utilized. The number of iterations for the dataset, often known as the epoch, was set at 20.

### Performance evaluation

The area under the ROC Curve (AUC) [23], accuracy, recall, precision, F1-Score and confusion matrix were used to evaluate classification performance.

### Experimental platform

This study conducts the implementation of a multi-view fusion model using Python (version 3.7.16) and TensorFlow (version 2.5.0). The experimental platform is a Lenovo server equipped with 32G of physical memory. The CPU is the Intel(R) Xeon(R) Silver 4210R CPU @ 2.40 GHz, while the graphics card model is the NVIDIA GeForce RTX 3080 Ti (12G). The operating system is the Ubuntu 18.04.6 LTS.

## Results

### Performance of the classification model

**Fig. 4:**
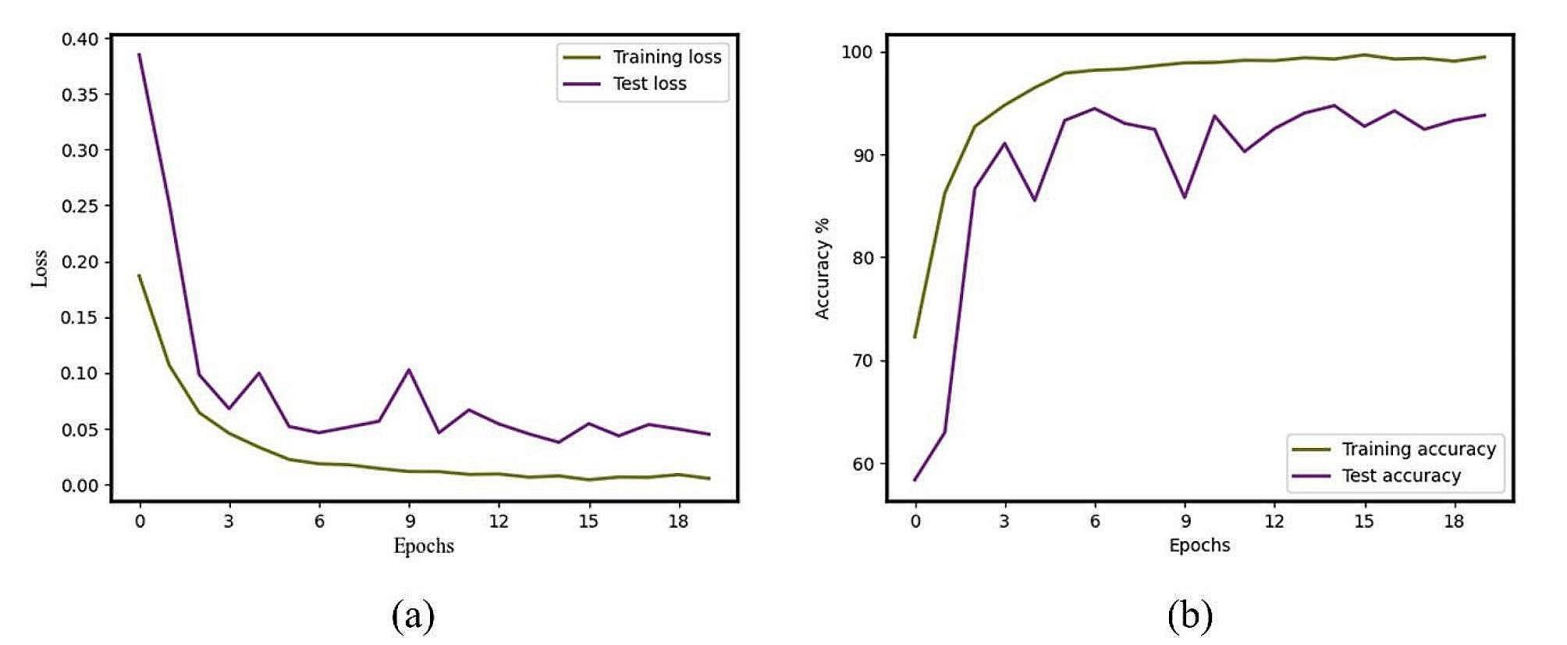
Loss and accuracy curves of our model over epochs in training and test. (**a**) Plot of multi-view fusion deep learning model loss function with number of iterations. (**b**) Plots of model accuracy as a function of the number of iterations in the training and test sets

**Fig. 5:**
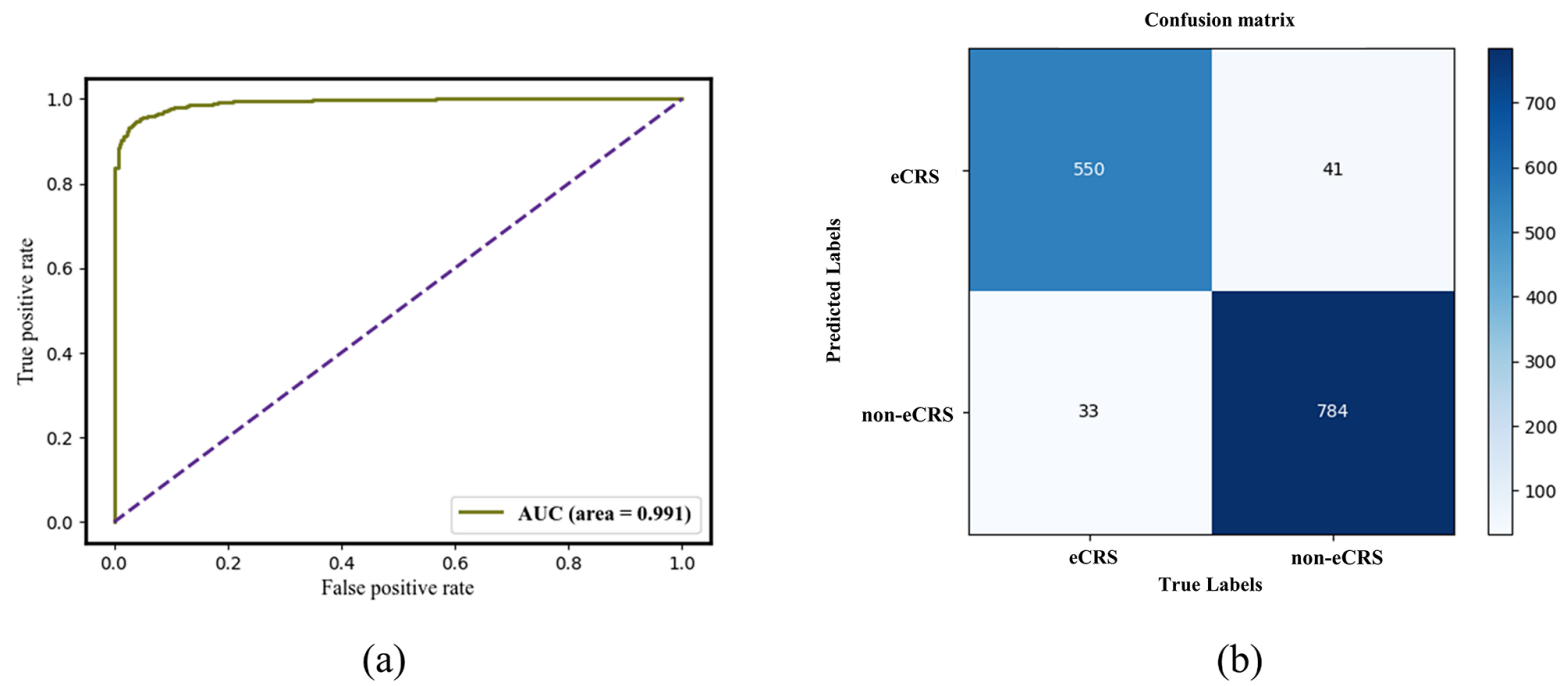
ROC curve and Confusion matrix of multi-view fusion model. (**a**) ROC curve, the area under curve is 0.991. (**b**) Confusion matrix of multi-view fusion model

The figures depicted in Fig. 4 illustrate the Loss and Accuracy curves of our model over Epoch in training and testing. The training Loss of the multi-view fusion classification model exhibits a decreasing trend in the training process, ultimately converging after 20 iterations. The model achieves a maximum accuracy of 96.54% on the test set at Epoch 14, so satisfying the inherent diagnostic accuracy criteria of the CRSwNP. Furthermore, the receiver operating characteristic (ROC) curve of the classifier is depicted in Fig. 5. It is worth noting that the area under the curve (AUC) attains a value of 0.991, providing evidence of the classifier’s commendable predictive capabilities. The Fig. 5 also illustrates the confusion matrix of the classifier’s classification results on the test set.

### Comparison with other models

**Fig. 6:**
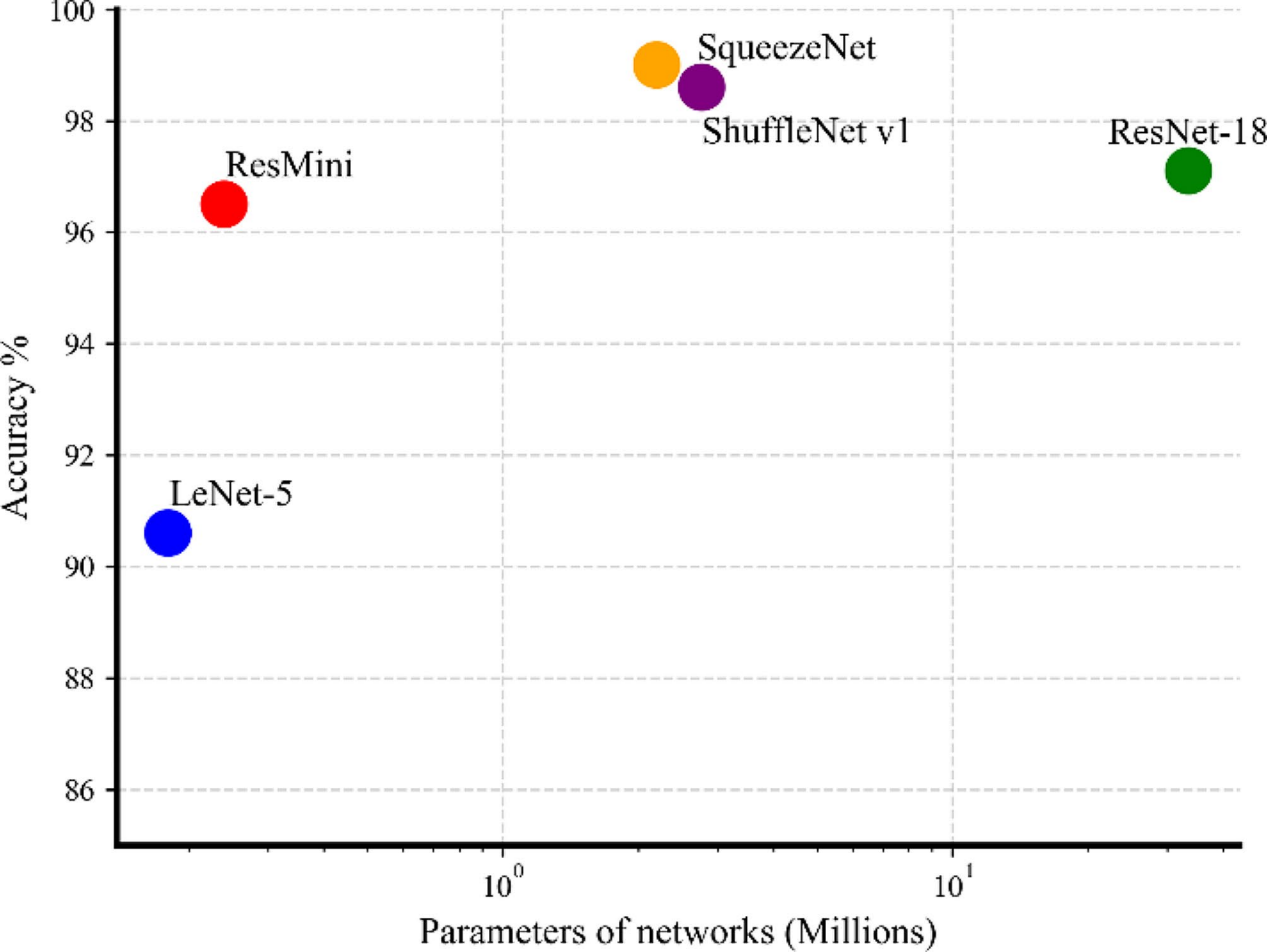
Comparison of the classification performance and network parameters of ResMini, ResNet-18 [13], LeNet-5 [24], ShuffleNet v1 [25], and SqueezeNet [26] on the sinus CT dataset. Please note that our Resmini can achieve a better balance between accuracy and parameter size

A comparison experiment was designed to further assess the proposed model. We substituted the Residual Block depicted in Fig. 2 with ResNet-18 [14], LeNet-5 [24], ShuffleNet v1 [25], and SqueezeNet [26] as shown in Fig. 6. In this context, we have retained the four feature extraction layers of the convolutional neural network, which includes components such as convolutional layer, pooling layer and batch normalization, and removed the classification layers, such as the fully connected layer. The feature extraction layer is used to generate a feature map containing the image features. Table 1 lists a summary of the test results obtained from the five networks when they were applied to the sinus CT test set. To minimize the effects of randomness, we employed 5-fold cross-validation by dividing the entire dataset into 5 equally sized segments. Subsequently, one segment was designated as the test set, while the remaining four segments were used for training. This process was repeated 5 times until each segment had been utilized as the test set once, with the others employed for training. The final evaluation metric value of the model was determined by averaging the evaluation metric values of the five iterations on the test data. The results indicate that ResMini exhibited a drop in accuracy of 2.5% when compared to SqueezeNet. However, the classification accuracy of 96.5% obtained by ResMini is a satisfactory outcome. Additionally, when compared to ResNet-18, ResMini experienced a decrease of accuracy 0.6%, which can be deemed as an acceptable outcome for our specific task. ResMini achieved a recall of 0.965 and a precision of 0.975 in the test set. In differentiating between types of intrinsic medical tasks, where each type is equally important, the F1-Score, a harmonic mean of precision and recall, serves as a comprehensive metric that accounts for both precision and recall. It is particularly applicable in scenarios with class imbalance. ResMini’s F1-Score reached 0.970, surpassing the other four models, indicating its superior performance in minimizing both false positives and false negatives. Regarding the AUC, ResMini is almost on par with SqueezeNet, achieving an impressive 0.991. A high AUC value generally indicates that the model performs exceptionally well in predicting positive and negative classes, offering high reliability and practicality, especially in handling complex or imbalanced datasets. Significantly, ResMini exhibits a notably lower parameter of 236,552, which is at least ten times smaller when compared to ResNet, SqueezeNet, and ShuffleNet v1. In similar studies, Du et al. utilized the ResNet-18 network to differentiate intrinsic features, but the study did not fully leverage the multi-angle CT scan data from the same patient, possibly leading to underutilization of information [13]. Our study conducted comparative experiments using a multi-view ResNet-18 network. The results demonstrate that ResMini maintains a similar accuracy to ResNet-18, while surpassing it in terms of precision and F1-Score. Moreover, the parameter count of the ResMini model is reduced by over 90% compared to ResNet-18, indicating that the original ResNet-18 network may have structural redundancies in this application scenario, necessitating optimization of the network architecture to learn more effective features.

**Table 1:** Evaluation metrics performance across models on dataset

Network	ResMini	ResNet-18	LeNet-5	ShuffleNet v1	SqueezeNet
Accuracy	96.5%	97.1%	90.6%	98.6%	99.0%
Recall	0.965	0.973	0.923	0.976	0.988
Precision	0.975	0.949	0.965	0.947	0.937
F1-Score	0.970	0.961	0.944	0.961	0.962
AUC	0.991	0.993	0.962	0.967	0.992
Total parameters (M)	0.24	33.54	0.18	2.77	2.21

## Discussion

The identification of CRSwNP endotyhpe is an essential issue in clinical medicine. Deep neural networks have significant potential to aid rhinologists in determining the endotype of CRSwNP. In conclusion, the implementation of this approach has the potential to alleviate the burden on rhinologists, enhance diagnostic efficacy, and offer appropriate and refined therapeutic treatment approaches for individuals with CRS.

This work also exhibits some shortcomings. Initially, data was exclusively obtained from a single hospital, and a standardized specialist database for CRSwNP was built. Further work is needed to expand the scope of the study by incorporating CT images of CRSwNP patients from various hospitals in China or perhaps globally to validate the model. It is imperative to gather extensive datasets from multiple centers in a prospective manner to effectively train and validate the artificial intelligence model.

Further advancements in artificial intelligence algorithms should be pursued to enhance the efficiency, convenience, and accuracy in diagnosing intrinsic form of CRSwNP. Furthermore, to address the complex nature of image data features in sinus CT, this study would develop a multi-view feature fusion network that incorporates an attention mechanism, so as to achieve adaptive focusing and fusion of key features across multiple views.

## Conclusions

This paper introduces a novel approach, wherein a multi-view deep learning fusion classification model is proposed for the purpose of diagnosing the endotype of CRSwNP using sinus CT scans. The multi-view perspective model effectively utilizes information by incorporating sinus CT axial, coronal, and sagittal image data, resulting in improved performance. From a computing resources standpoint, the model aims to minimize the number of parameters while maintaining a satisfactory level of accuracy. This approach effectively conserves computer resources and makes it ideal for integration into medical diagnostic and treatment equipment.

## Data Availability

The data used for the analysis are available from the corresponding authors upon request.
